# 1126. Case Study. Moving Forward: Evaluating the Safety of Mobilizing Burn Patients on ECMO

**DOI:** 10.1093/jbcr/irag033.548

**Published:** 2026-04-06

**Authors:** Catherine Freeman, Jillian Smith, Victoria Allen

**Affiliations:** University of Cincinnati Medical Center, Cincinnati, OH; University of Cincinnati Health, Cincinnati, OH; University of Cincinnati Medical Center, Cincinnati, OH

## Abstract

**Patient Presentation (age range, injury details, relevant history):**

Patient is a 33 year old who sustained 50% TBSA burns. Following allografting he was extubated but developed acute respiratory distress syndrome and required re-intubation. Despite the patient showing improvement in oxygenation, his ventilator settings remained high, the patient was placed on veno-venous extracorporeal membrane oxygenation (VV-ECMO). The patient was cannulated through his right internal jugular and femoral vein.

**Clinical Challenges:**

Clinical challenges faced by the burn therapists included the appropriateness of the patient for mobilization and range of motion (ROM) on VV ECMO and inexperience with mobilization of this patient population. It was determined with the medical team that the patient was safe to be mobilized on VV ECMO. Prior to sitting the patient on the side of the bed, the head of bed was elevated above 50* to assess flow stability before mobilizing the patient. ROM restrictions were placed for right shoulder abduction and flexion and right hip flexion limiting motion to 90* to prevent kinking or dislodging of the cannula. Due to the need for VV ECMO, the patient was transferred to the Cardiovascular Intensive Care Unit. Mobilization activities were carried out by the ECMO trained therapists with burn therapists present during the session to make recommendations to optimize cutaneous functional units. Since this case, the burn therapy team has received training on mobilization of ECMO patients.

**Management Approach:**

While on VV ECMO, the patient was approved by the team to participate in therapy. The patient received therapy twice daily with one session focused on ROM and the other for mobility. Sessions involving ROM were conducted by an ECMO trained therapist with the ECMO specialist. To prevent decreases in flow rate and cannula kinking, range of motion was restricted to 90 degrees of shoulder flexion and abduction and 90 degrees of hip flexion. Mobility sessions required the ECMO trained therapist, RT, RN, and the ECMO specialist. The patient was able to participate in 12 therapy sessions, 10 focused on ROM and 2 mobility sessions. During the two mobility sessions, he was able to sit edge of bed to complete trunk exercises and stand.

**Outcomes:**

12 therapy sessions were completed with no adverse events reported. The ECMO circuit and flows were stable throughout all sessions. Cannula insertion sites were assessed pre and post therapy. For 10 of the 12 therapy sessions the cannula site were noted to be intact and stable pre and post therapy. On one session, it was noted that the cannula anchor required adjustment by ECMO specialist. In the other session, there was oozing from the right internal jugular vein which remained unchanged. The patient was admitted for 46 days and discharged home.

**Lessons Learned:**

Lessons that we learned from this case is that ECMO is not a barrier to mobility or ROM for burn patients. Depending on the cannula site, ROM of a limb should be restricted to prevent decannulation, prevent cannula kinking and subsequent decreases in flow.

